# Preparation of Functionalized Zr-Based MOFs and MOFs/GO for Efficient Removal of 1,3-Butadiene from Cigarette Smoke

**DOI:** 10.3390/ma16020684

**Published:** 2023-01-10

**Authors:** Yunxin Yang, Cong Wang, Hua Zhang, Jiancai Qian, Song Yang, Huiyun Liao, Xuehui Sun, Yipeng Wang, Peijian Sun, Yunzhen Jia, Junwei Guo, Huaiyuan Zhu, Cong Nie

**Affiliations:** Zhengzhou Tobacco Research Institute of CNTC, Zhengzhou 450001, China

**Keywords:** UiO-66, UiO-66-NH_2_/GO, 1,3-butadiene removal, cigarette smoke

## Abstract

Removal of 1,3-butadiene from cigarette smoke plays an important role in human health and environmental protection. Herein, a series of UiO-66 X% containing different ratios of the -NH_2_ group was synthesized via the solvothermal method by using terephthalic acid (H_2_BDC) and 2-aminoterephthalic acid (NH_2_-BDC) as ligands. Using GO as support, a series of UiO-66-NH_2_/GO Y% were prepared by controlling the ratio of UiO-66-NH_2_ and GO. The effects of -NH_2_ and GO contents on the structure and composition of MOFs were investigated. Finally, the different -NH_2_ contents of UiO-66 X% and the different GO contents of UiO-66-NH_2_/GO Y% were applied in 1,3-butadiene removal from cigarette smoke. The results showed that UiO-66 X% with the higher contents of -NH_2_ showed a higher rate of 1,3-butadiene removal, and UiO-66-NH_2_/GO Y% with the GO contents of 5% showed the highest removal rate of about 33.85%, which was 25.54% higher than that of activated carbon. In addition, the saturation capacity of the adsorbent materials for 1,3-butadiene was as high as 210.01–239.54 mg/g, showing great potential in reducing harmful components in cigarette smoke and environmental protection.

## 1. Introduction

Cigarette smoke is a complex aerosol containing more than 7000 components, of which about 1% have carcinogenic or potentially hazardous effects on humans [1,2]. The harmful components mainly include NH_3_, HCN, TSNA, low-weight aldehydes, and hazardous volatile organic compounds (VOCs) [3]. Among those harmful components, 1,3-butadiene was not only classified as a Group 1 carcinogen (definite carcinogen) by the International Agency for Research on Cancer (IARC) but also listed as one of the 18 priority regulatory components of cigarette smoke by the World Health Organization (WHO, Geneva, Switzerland) [4,5]. Excessive inhalation of 1,3-butadiene in a short time will lead to dizziness, headache, and burn in the nasal cavity, throat, and trachea. Long-term exposure to 1,3-butadiene causes irreversible damage to humans and significantly increases the risk of cancer [6,7]. Due to the severe toxicity and the high release of 1,3-butadiene in cigarette smoke (~40 μg·cig^−1^) [8,9], the efficient removal of 1,3-butadiene from cigarette smoke has significant implications for human health and environmental protection.

Currently, the adsorption of 1,3-butadiene is mainly studied in petrochemical and rubber production [10], and the adsorption materials are especially activated carbon, zeolites, porous polymer resin, and metal–organic frameworks (MOFs) [11,12]. Guillaume B. Baur et al., prepared the modified Na-X-H_2_O zeolite using soft hydrothermal treatment. The treatment removed the Al^3+^ in the material, leaving behind new OH-groups which significantly increased the adsorption capacity of 1,3-butadiene [13]. Zhang et al., synthesized several isomeric anion-pillared interpenetrated hybrid ultramicroporous materials which could separate and purify 1,3-butadiene from C_4_ olefins effectively. Their synthesis method could excellently control the pore size and functional sites of the material by altering the anionic column and organic linkers [14]. Albert M. Tsybulevski et al., prepared a transition metal polycation exchange zeolite by ion exchange of octahedral zeolites with the corresponding salt solution under hydrolytic conditions. The presence of transition metals increased the acid center sites and π-complexation effect of the zeolites, resulting in a higher adsorption capacity for 1,3-butadiene than mono-cationic zeolites [15]. Ihtisham et al., synthesized the bimetallic MOF (Ni-ZIF-8) to successfully separate pure 1,3-butadiene from the 1,3-butadiene/N_2_ mixture, which had higher adsorption capacity and selectivity than zeolites [16]. Keisuke Kishida et al., synthesized the SD-65 with a unique gate-opening phenomenon. SD-65 had a triple interpenetrating framework structure, therefore 1,3-butadiene could change the structure of the framework due to its more significant quadrupole moment, leading to the highly selective adsorption of 1,3-butadiene in C_4_ mixtures [17]. Many advances have been made in the adsorption of 1,3-butadiene. However, the removal of 1,3-butadiene from cigarette smoke is rarely reported.

Cigarette smoke as a unique system is usually characterized by complex components, large aerosol particle diameter (0.1 μm–1.0 μm), high concentration, and short contact time with the adsorption medium (0.01 s) [18]. Based on these properties of cigarette smoke, adsorbents for removing 1,3-butadiene from cigarette smoke should have these characteristics. (I) Rich pore structure for rapid transport of cigarette smoke and adsorption of 1,3-butadiene. (II) High specific surface area for adequate contact between cigarette smoke and adsorbents. (III) Excellent structural stability for maintaining activity in a cigarette smoke environment. (IV) π-conjugated structure for π-π conjugation with 1,3-butadiene. UiO-66 is a class of MOFs with the highest metal cluster–ligand coordination bond, its structural unit consists of [Zr_6_O_4_(OH_4_)] metal cluster linked to 12 H_2_BDC ligands. The strong π-conjugation effect and rich pore structure are the main mechanisms of the adsorption of 1,3-butadiene from cigarette smoke. The excellent stability ensures that UiO-66 will maintains activity and structural stability in the cigarette smoke environment [19,20,21]. Graphene oxide (GO), the product of the chemical exfoliation of graphite, and the abundant oxygen-containing functional groups on its surface offer the possibility of combining with MOFs. The MOFs/GO prepared by combining MOFs and GO not only has a rich pore structure but also has a lamellar structure that facilitates the passage of macromolecules in cigarette smoke [22,23], which is expected to achieve efficient removal of 1,3-butadiene from cigarette smoke.

In this study, a series of UiO-66 X% with different -NH_2_ contents were prepared via the solvothermal method by controlling the ratio of H_2_BDC and NH_2_-BDC. A series of UiO-66-NH_2_/GO Y% with different GO contents were prepared by controlling the GO contents. The effects of the contents of -NH_2_ and GO on the structure and composition were investigated. Finally, the removal of 1,3-butadiene from cigarette smoke by using UiO-66 X% and UiO-66-NH_2_/GO Y% as absorbents was investigated.

## 2. Materials and Methods

### 2.1. Materials

ZrCl_4_ (99.5%), graphite, NaNO_3_, H_2_BDC, NH_2_-BDC, N,N-dimethylformamide (DMF), H_2_SO_4_ (98%), KMnO_4_, H_2_O_2_, HCl, and activated carbon were purchased from Aladdin Chemical Reagent Co., Ltd. 1,3-Butadiene standard (2.0 mg/mL in MeOH) was purchased from Beijing Bailingway Technology Co., Ltd. (Beijing, China). Methanol, ethanol, isopropanol (HPLC), and D_6_-benzene (AR) were purchased from Sigma-Aldrich. Cigarettes were provided by China National Tobacco Corporation.

### 2.2. Synthesis Methods

#### 2.2.1. Synthesis of MOFs

UiO-66 X% (X is the NH_2_-BDC molar fraction): UiO-66 X% was synthesized by the solvothermal method [21,24]. ZrCl_4_ (3 mmol) was used as the metal source and H_2_BDC and NH_2_-BDC (3 mmol total) were used as ligands, and the contents of NH_2_-BDC were controlled at 0%, 25%, 50%, 75%, and 100% (preparation parameters are listed in Table 1). The mixture was sonicated in DMF (50 mL) until completely dissolved. Subsequently, the mixture was added to a 100 mL PTFE-lined stainless steel autoclave and placed in a preheated oven at 120 °C for 24 h. When the reaction was over, and the autoclave was cooled to room temperature, the product was washed with DMF and anhydrous ethanol three times. Then, the white product was put into the oven at 100 °C for 12 h. Subsequently, a soxhlet extraction was performed at 100 °C for 48 h with anhydrous ethanol as the solvent to remove unreacted impurities from the product completely. Finally, the product was washed with deionized water to remove the residual solvent and impurities thoroughly. The product was obtained after drying in the oven.

#### 2.2.2. Synthesis of GO

GO was prepared via the modified Hummers method [25,26]. Graphite (2 g) and NaNO_3_ (2 g) were placed in a three-neck flask and stirred. Then, 90 mL of H_2_SO_4_ (98%) was added into the ice bathtub system, and the temperature was maintained at 0–5 °C. KMnO_4_ (10 g) was gradually added to the system and reacted for 1 h. Subsequently, the product was transferred to a three-neck flask in a water bathtub at 35 °C with continuous stirring. When the system temperature reached 35 °C, 184 mL of warm ultrapure water was slowly added and stirred for 2 h. Then the system temperature was raised to 98 °C and continuously stirred for 2 h in the water bathtub. Next, 40 mL of H_2_O_2_ (30 wt%) and 200 mL of water were added to remove residual KMnO_4_. After that, the solution was centrifuged to collect the precipitation at 10,000 rpm for 10 min. Then the precipitation was redispersed into a solution of hydrochloric acid (10%) and deionized water. The above processes were repeated three times to obtain the target product. Finally, the product was vacuum-dried overnight for further use.

#### 2.2.3. Synthesis of UiO-66-NH_2_/GO Y%

UiO-66-NH_2_/GO Y% (Y is m_(GO)_:m_(ZrCl4)_): GO was added to 40 mL of DMF and sonicated for 3 h until GO was completely dissolved. Subsequently, ZrCl_4_ and NH_2_-BDC were added, and sonication was continued for 30 min until it was wholly dissolved (preparation parameters are listed in Table 1). The mixture was then placed in a 100 mL PTFE-lined stainless steel autoclave and placed in a preheated oven at 120 °C for 24 h, and a series of UiO-66-NH_2_/GO Y% with different GO contents were obtained by following the method of synthesizing the UiO-66 X% [27].

### 2.3. Characterization of Materials

Fourier transform infrared spectroscopy (FT-IR) was measured on a Bruker Tensor 27 Spectrometer using the KBr method in the range of 4000–400 cm^−1^ to analyze the chemical bonds and functional groups of materials. Elemental analysis (EA) was obtained by the Organic Element Analyzer (Elementar, Langenselbod, Germany). X-ray diffraction patterns (XRD) were obtained by the X-ray diffractometer (Ultima IV, Tokyo, Japan) with a scanning range of 5–90° and a scanning speed of 2°/min. The structure and crystallinity of crystals were reflected by their position and intensity of diffraction peaks. N_2_ adsorption/desorption analysis was measured to be 77.3 K using a Micrometrics Tristar II 3020 surface area and porosity analyzer (Norcross, GA, USA). Before analysis, the samples were outgassed at 100 °C for 12 h under a vacuum. The specific surface area was calculated by the standard Brunauer–Emmet–Teller (BET) equation. The micropore volume was derived from the t-polt method, and the pore size distribution was obtained from the nonlocal density functional theory (NLDFT) model. The field emission scanning electron microscopic (SEM) images were taken by a field emission electron scanning microscope (ZEISS Gemini 300, Oberkochen, Germany) to observe the morphology and structure of the materials.

### 2.4. Cigarette Smoke Adsorption Experiment

**Figure 1:**
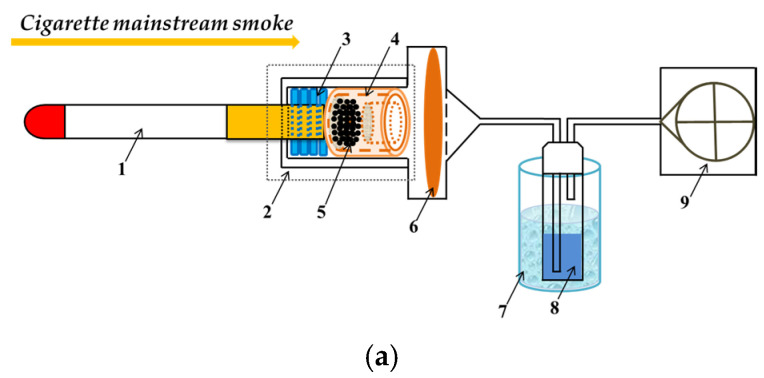

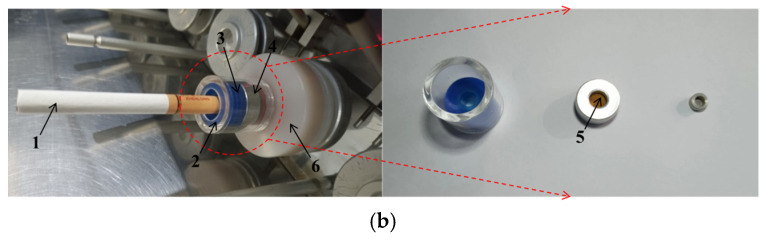
(**a**) Illustration for cigarette smoke adsorption (1: cigarette; 2: homemade device; 3: rubber ring; 4: device for loading adsorbent material; 5: adsorbent material; 6: the particle phase trap with Cambridge filter pad; 7: cold trap containing isopropanol and dry ice; 8: absorption flask with 15 mL of methanol; 9: smoking machine). (**b**) Actual diagram of cigarette smoke adsorption device.

The removal of 1,3-butadiene from cigarette smoke using these adsorbent materials was evaluated on a homemade device linked to the smoking machine (Figure 1). The cigarette has a circumference of 24.4 mm and a length of 84 mm. Cigarettes with a mass of 975 ± 15 mg and draw resistance of 1030 ± 30 Pa were selected, and equilibrated under a constant temperature of 22 °C and relative humidity of 60% for 48 h. The ISO standard smoking pattern was adopted with a puff volume of 35 mL, puff duration of 2 s, and puff interval of 60 s. The added amounts of adsorbents were 10 mg/cig and 4 cigs of parallel smoking. Cigarette mainstream smoke was generated from the combustion cone and passed through the adsorbents packing area for adsorption. The total particulate matter (TPM) was intercepted by the Cambridge filter pad, and 1,3-butadiene in the gas phase of the cigarette smoke was collected by the absorption flask filled with methanol in the cold trap.

The yield of 1,3-butadiene in the smoke was determined by the GC-MS coupled instrument (Agilent 5977A) with the standard internal method (D*_6_*-benzene as internal standard). The standard curve of 1,3-butadiene was plotted as y = 2546.0786x − 1.4674 (x indicates the ratio of 1,3-butadiene peak area to D*_6_*-benzene peak area, y indicates the mass of 1,3-butadiene (μg)). The removal rate of 1,3-butadiene in cigarette smoke was determined by the comparison of the control cigarettes (no materials added) and the test cigarettes (prepared materials added).

### 2.5. Adsorption Experiments of 1,3-Butadiene

The adsorption isotherms of pure 1,3-butadiene were measured using Micromeritics ASAP-2460 and the adsorption amount was determined at each pressure point. About 250 mg of adsorbents was placed into the adsorption tube and degassed under vacuum at 80 °C for 12 h. Subsequently, 1,3-butadiene adsorption experiments were performed under the condition of 298 K and the relative pressure (P/P_0_) from 0 to 1. The amount of 1,3-butadiene adsorbed was determined after reaching adsorption equilibrium.

## 3. Results and Discussion

### 3.1. Influence of -NH_2_ Contents on the Structure and Composition of UiO-66 X%

**Figure 2:**
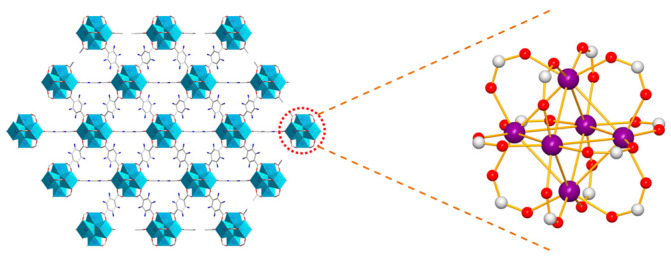
Schematic diagram of the internal structure of UiO-66. Purple, red, and white represent Zr, O, and C, respectively.

Figure 2 shows the crystal structure of UiO-66. The metal clusters with an octahedral structure were formed with six Zr atoms. The structural unit of UiO-66 was assembled by coordination of the metal cluster with 12 H_2_BDC through Zr-O bonding. UiO-66 with a periodic reticular structure was obtained by the continued extensional growth of the structural unit. A series of UiO-66 X% were obtained by changing the ratio of NH_2_-BDC and H_2_BDC.

**Figure 3:**
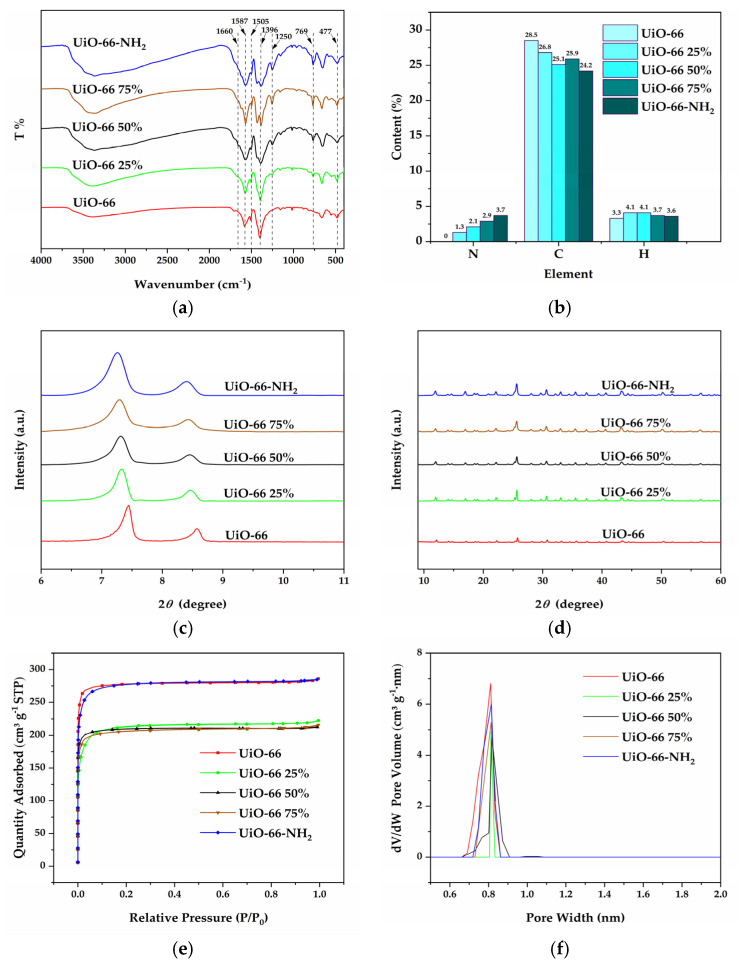
FTIR spectrum (**a**) and elemental contents (**b**) and XRD pattern (**c**,**d**) and N_2_ adsorption–desorption isotherms curves (**e**) and pore size distributions (**f**) of UiO-66 X%.

Figure 3a shows the FTIR spectrum of UiO-66 X%. For UiO-66, the peak at 1660 cm^−1^ was considered the C=O stretching vibration peak of carboxylic acid in H_2_BDC. The peaks at 1587 cm^−1^ and 1396 cm^−1^ were identified as the asymmetric stretching vibration and the symmetric stretching vibration of O-C-O in H_2_BDC. The peak at 1505 cm^−1^ was attributed to the stretching vibration of C=C in the benzene ring. The characteristic Zr-O bond stretching vibration peak was observed at 477 cm^−1^, indicating the successful assembly of UiO-66 [19,28]. The peaks at 769 cm^−1^ and 1250 cm^−1^ corresponded to the stretching vibration of N-H and C-N [29,30], and the peak enhanced with the increase in NH_2_-BDC contents, indicating the presence of -NH_2_. To further demonstrate the successful introduction of NH_2_-BDC, the elemental contents plot (Figure 3b) showed that UiO-66 had no N element, while the N element contents of UiO-66 X% increased significantly with the increase in NH_2_-BDC contents, which proved the successful introduction of NH_2_-BDC.

Figure 3c,d showed the XRD pattern of UiO-66 X%. For UiO-66, characteristic peaks at 2θ angles of 7.44°, 8.57°, 12.07°, 14.19°, 14.80°, 17.13°, 22.28°, 25.77°, 33.15° corresponded to the (111), (002), (022), (113), (222), (004), (115), (006), and (137) crystal planes, respectively. The positions of the diffraction peaks were highly consistent with those reported in the literature [21]. All of the UiO-66 X% had characteristic peaks and high resolution consistent with UiO-66, which showed that the UiO-66 X% with the introduction of NH_2_-BDC still maintained a high degree of crystallinity. The prominent feature peak on the (111) crystal plane indicated the excellent orientation of UiO-66 X% [19]. Figure 3c showed a slight leftward shift of the diffraction peak of UiO-66 X% with increasing NH_2_-BDC contents, which was probably attributed to the cell expansion caused by the -NH_2_ groups. The introduction of -NH_2_ groups raised the crystal plane spacing (d), and the diffraction angle (θ) decreased accordingly, as shown by the Bragg equation 2dsinθ = nλ [31,32].

Figure 3e displayed the nitrogen adsorption/desorption isotherms and pore size distributions of UiO-66 X%. They exhibited typical type I isotherms according to the IUPAC classification. The adsorption of N_2_ increased sharply in the lower range of relative pressure (P/P_0_). In comparison, almost no N_2_ was adsorbed in the higher relative pressure range, indicating that UiO-66 X% mainly exhibited the microporous structure. For UiO-66 X%, UiO-66 and UiO-66-NH_2_ obtained by using H_2_BDC and NH_2_-BDC as single ligands had the large BET surface area and pore volume, and UiO-66 had the largest BET surface area of 1135 m^2^/g and pore volume of 0.43 cm^3^/g (Table 2). In contrast, UiO-66 X% obtained using H_2_BDC and NH_2_-BDC as mixed ligands showed varying degrees of reduction in BET surface area and pore volume. The possible reason was that the mixed ligands affected the orderliness of the UiO-66 X% structure and caused crystal defects resulting in lower BET surface area and pore volume [32]. The pore size distribution (Figure 3f) showed that all of the UiO-66 X% had a consistent pore size of about 0.81 nm, which indicated that the introduction of NH_2_-BDC had no effect on the pore size of UiO-66 [32].

**Figure 4:**
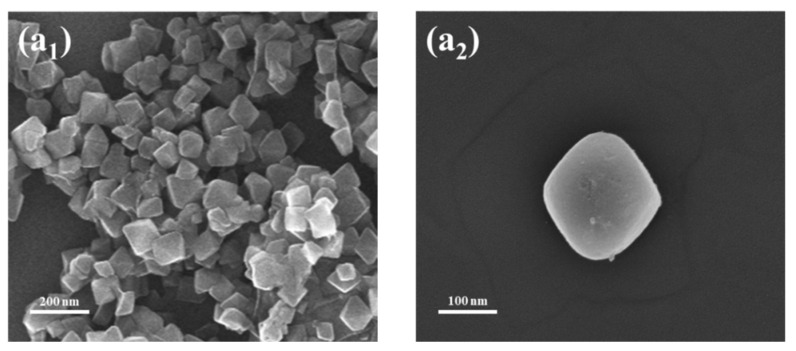

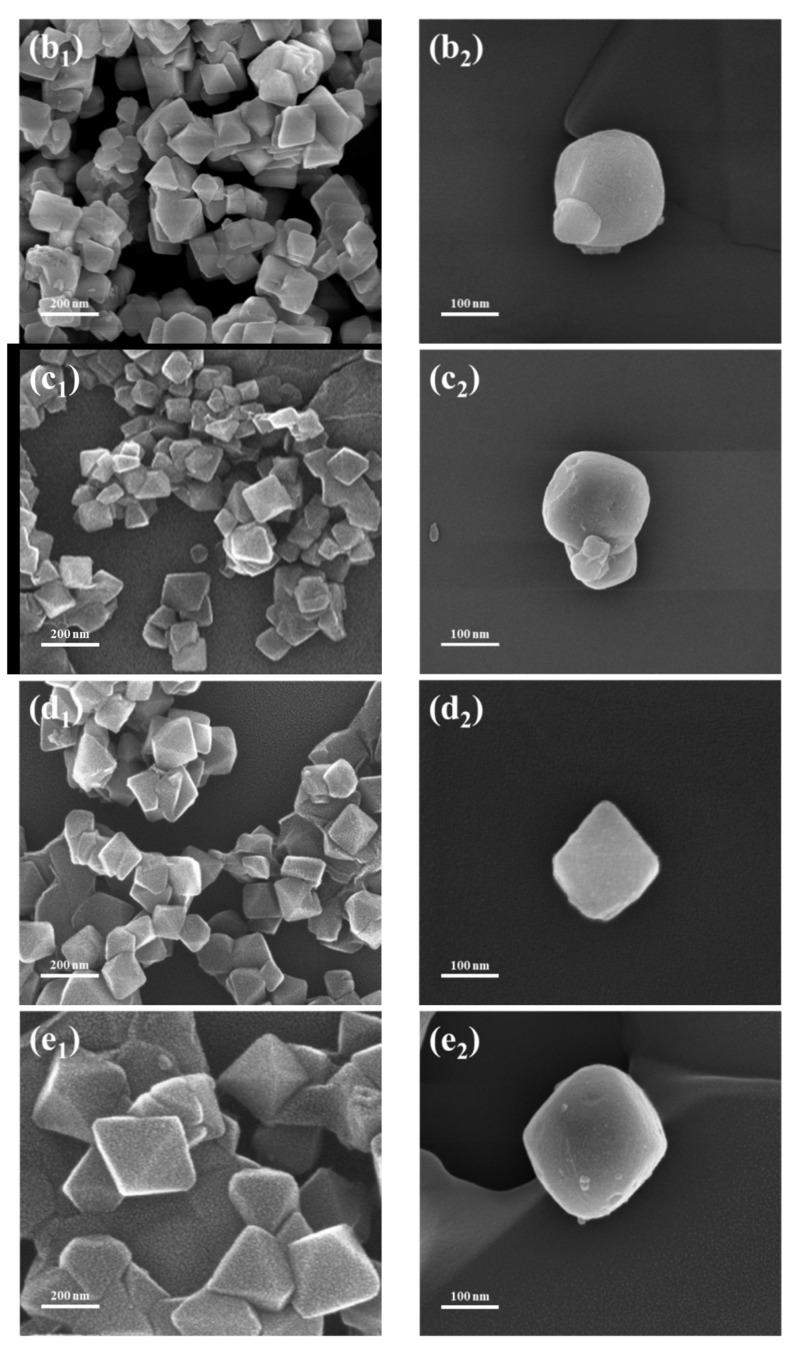
SEM of (**a_1_**) UiO-66 and (**b_1_**) UiO-66 25% and (**c_1_**) UiO-66 50% and (**d_1_**) UiO-66 75% and (**e_1_**) UiO-66-NH_2_ at lower magnification; SEM of (**a_2_**) UiO-66 and (**b_2_**) UiO-66 25% and (**c_2_**) UiO-66 50% and (**d_2_**) UiO-66 75% and (**e_2_**) UiO-66-NH_2_ at higher magnification.

Figure 4 showed the SEM images of UiO-66 X%, in which UiO-66 and UiO-66-NH_2_ exhibited the perfect ortho-octahedral structure with a crystal diameter of about 150–200 nm, consistent with the literature report [30]. While the UiO-66 X% using mixed organic ligands still exhibited the stable octahedral structure, the crystal morphology and structure appeared significantly different with a reduced degree of regularity. This could be related to the crystal defects caused by the mixed ligands.

### 3.2. Influence of GO Loading on the Structure and Composition of UiO-66-NH_2_/GO Y%

**Figure 5:**
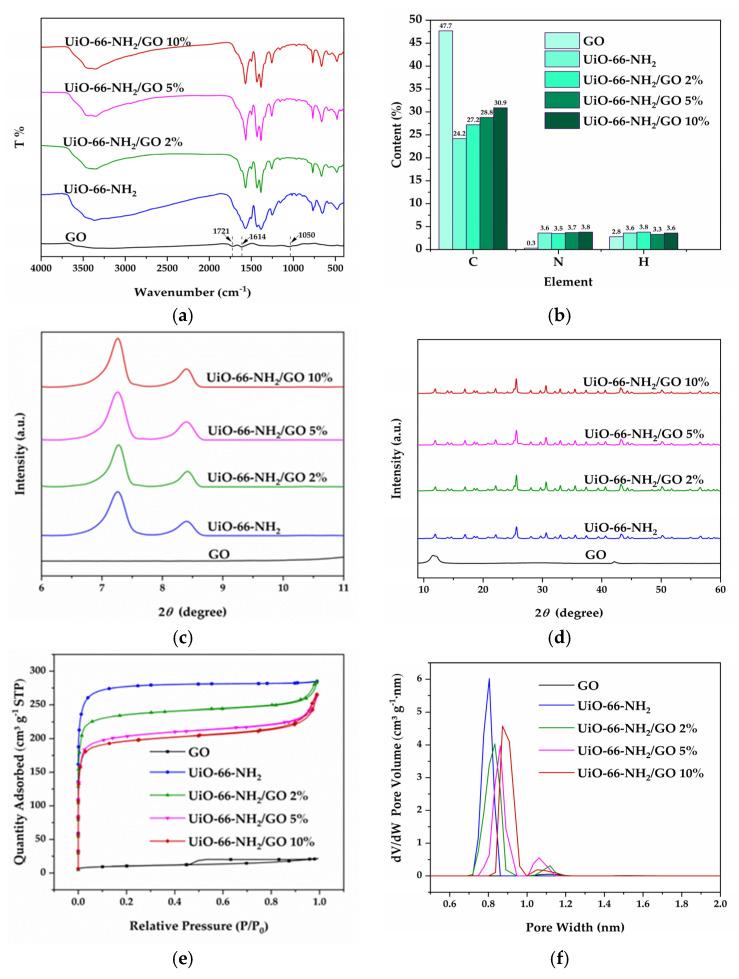
FTIR spectrum (**a**) and elemental contents (**b**) and XRD pattern (**c**,**d**) and N_2_ adsorption–desorption isotherms curves (**e**) and pore size distributions (**f**) of UiO-66-NH_2_/GO Y%.

GO has a laminar structure that facilitates the passage of macromolecules in cigarette smoke. To improve the removal of 1,3-butadiene yield, a series of UiO-66-NH_2_/GO Y% containing different GO contents were constructed. Figure 5a showed the FTIR spectra of UiO-66-NH_2_/GO Y%. For GO, the peaks at 1721 cm^−1^, 1614 cm^−1^, and 1050 cm^−1^ were considered as the C=O stretching vibration peak, C=C stretching vibration peak, and C-O stretching vibration peak, respectively [22]. The characteristic absorption peaks of partial oxygen-containing functional groups in GO decreased or even disappeared in the spectra of the composites, owing to the coordination between the carboxyl groups of GO and Zr^4+^ [33]. To further demonstrate the successful composite of UiO-66-NH_2_ and GO, the elemental contents plot (Figure 5b) showed that the C content in GO was as high as 47.7%, and the C contents in the composites were significantly higher than that in UiO-66-NH_2_, and the C contents increased significantly with the increased of GO contents, which proved the successful compound of UiO-66-NH_2_ and GO.

Figure 5c,d showed the XRD pattern of UiO-66-NH_2_/GO Y%. For GO, the broad XRD characteristic peak at 11.60° reflected the layered structure of GO. The UiO-66-NH_2_/GO Y% had consistent characteristic peaks with UiO-66-NH_2_, which showed that UiO-66-NH_2_ in the composites still maintained a high degree of crystallinity. In addition, the loading UiO-66-NH_2_ promoted the layer exfoliation of GO, but GO still maintained layer structure, in fact. For UiO-66-NH_2_/GO Y%, the diffraction peaks of GO layered structure and UiO-66-NH_2_ at around 11.60° were overlapped [34,35].

Figure 5e displayed the nitrogen adsorption/desorption isotherms and pore size distributions of UiO-66-NH_2_/GO Y%. The isotherms showed that GO had minimal adsorption capacity for N_2_, especially since there was almost no absorption of N_2_ in the lower relative pressure range, which was attributed to the lamellar structure of GO. The composites obtained by introducing GO had a smaller BET surface area and pore volume than UiO-66-NH_2_, and the BET surface area and pore volume decreased with the increase in GO contents. The BET surface area decreased to 928 m^2^/g, 794 m^2^/g, and 766 m^2^/g, and the pore capacity decreased to 0.35 cm^3^/g, 0.32 cm^3^/g, and 0.29 cm^3^/g when the GO contents were 2%, 5%, and 10%, respectively, which was caused by the inclusion of non-porous GO [36]. Compared to UiO-66-NH_2_, the composites absorbed N_2_ in the higher relative pressure range. The pore size distribution (Figure 5f) showed that UiO-66-NH_2_ had only a single microporous structure with a size of 0.81 nm, and GO contained a few pore structures of 1.12 nm. When the GO contents were 2%, 5%, and 10%, the pore size increased to 0.83 nm, 0.86 nm, and 0.91 nm, respectively, and a small amount of pore structure also appeared at 1.12 nm and 1.06 nm (Table 3). This was attributed to the bonding of Zr^4+^ with the oxygen-containing functional groups on the GO surface, resulting in a larger pore structure [33].

**Figure 6:**
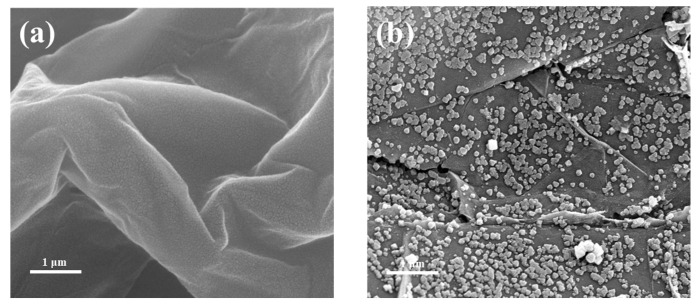

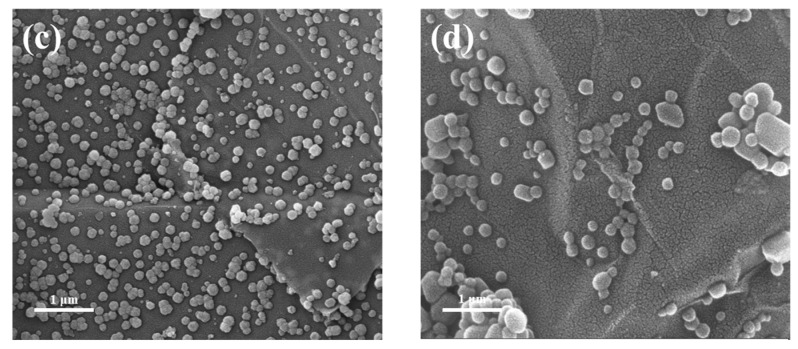
SEM of (**a**) GO and (**b**) UiO-66-NH_2_/GO 2% and (**c**) UiO-66-NH_2_/GO 5% and (**d**) UiO-66-NH_2_/GO 10%.

Figure 6 showed the SEM images of UiO-66-NH_2_/GO Y%. GO exhibited a perfect lamellar structure, and the composites exhibited that UiO-66-NH_2_ was distributed on the GO surface. When the GO content was 2%, UiO-66-NH_2_ was relatively aggregated and adhered to the GO sheet. When the GO content was 5%, UiO-66-NH_2_ was orderly and uniformly dispersed on the GO sheet. When the GO content reached 10%, UiO-66-NH_2_ was sparsely and unevenly distributed on the GO sheet, and the crystal structure of UiO-66-NH_2_ decreased in regularity. The reason was that too many Zr metal atoms combined with the surface oxygen-containing functional groups of GO affected the crystallization of UiO-66-NH_2_ [33].

### 3.3. Performance Evaluation of 1,3-Butadiene Removal from Cigarette Smoke

1,3-Butadiene, one of the 18 priority toxicants in cigarette smoke proposed by the WHO, has a significant impact on the health of humans. To evaluate the rate of 1,3-butadiene removal in cigarette smoke, UiO-66 X% and UiO-66-NH_2_/GO Y% were used as the adsorbents and cigarette smoke was trapped in the cold trap by 15 mL of methanol solution. The content of 1,3-butadiene in cigarette smoke was calculated from the standard curve of 1,3-butadiene, and the removal rate was calculated by comparing control cigarettes with experimental cigarettes. The effect of the contents of -NH_2_ and GO on the performance of UiO-66 X% and UiO-66-NH_2_/GO Y% was analyzed by comparing the removal effect. As a comparison, the effectiveness of activated carbon as an adsorbent for the removal of 1,3-butadiene was evaluated.

Table 4 showed the amount and removal rate of 1,3-butadiene released from cigarette smoke. The yield of 1,3-butadiene in cigarette smoke was 38.12 μg·cig^−1^ without the adsorbents. There was only an 8.31% removal rate of 1,3-butadiene from cigarette smoke using activated carbon as the adsorbent. However, the removal rate of 1,3-butadiene by UiO-66 was 11.15% under the same amount of adsorbent addition. In comparison, the adsorption of UiO-66 was significantly improved than that of activated carbon. This might be attributed to the π-π conjugate action between UiO-66 and 1,3-butadiene molecules. For UiO-66 X%, the removal performance of 1,3-butadiene gradually improved with the increasing contents of NH_2_-BDC. The removal effect of UiO-66 X% increased from 11.15% to 13.71% when the NH_2_-BDC contents increased from 0% to 100%. The reason was that the presence of -NH_2_ activated the carboxyl groups at both ends of NH_2_-BDC, causing the breakage of the Zr-O bond and exposing more Zr metal active sites [32]. The π-complexation between the double bond of diene and the exposed metal atom increased the driving force which encouraged the movement of the 1,3-butadiene to the adsorption site [15,37]. Thus, it increased the adsorption capacity for 1,3-butadiene. For all UiO-66 X% containing NH_2_-BDC, the specific surface area and pore volume were smaller than UiO-66, indicating that the adsorption of 1,3-butadiene by UiO-66 X% was not only dependent on physical adsorption based on the specific surface area and pore volume, but also on chemisorption based on the interaction between UiO-66 X% and 1,3-butadiene molecules. However, all of the UiO-66 X% had limited removal of 1,3-butadiene. The possible reason was that the single pore structure of UiO-66 X% was unfavorable for the smooth passage of complex components in cigarette smoke, limiting the effective adsorption of 1,3-butadiene.

When using pure GO as the adsorbent, only 7.06% of 1,3-butadiene was removed from cigarette smoke. This was because the lamellar structure of GO was unfavorable for the effective trapping of the components in cigarette smoke. The composites UiO-66-NH_2_/GO Y%, obtained by combining UiO-66-NH_2_ and GO, significantly improved the removal rate of 1,3-butadiene from cigarette smoke. The results showed that UiO-66-NH_2_/GO Y% could effectively remove 26.73–33.85% of 1,3-butadiene, which was substantially higher than that of UiO-66-NH_2_ and activated carbon. The primary reason was that the composites obtained the unique lamellar structure of GO, which could compensate for the limitations of the single microporous structure. The interlayer channels of GO facilitated the smooth passage of the complex components in cigarette smoke, and the microporous structure and π-π conjugation of UiO-66-NH_2_ facilitated the trapping of 1,3 butadiene molecules. Therefore, the composites had a higher rate of 1,3-butadiene removal.

Comparing the effectiveness of UiO-66-NH_2_/GO Y% with different GO contents in removing 1,3-butadiene from cigarette smoke. When the GO content was 2%, the removal rate of 1,3-butadiene from cigarette smoke was 26.73%, which was significantly higher than that of UiO-66-NH_2_ and activated carbon. However, too little GO content resulted in the dense distribution of UiO-66-NH_2_ on the GO surface. The removal rate was 28.14% when the GO content was 10%. However, UiO-66-NH_2_ was sparsely distributed on the surface of GO because of the large amount of GO doping, and the combination of a large amount of Zr metal with oxygen-containing functional groups on the surface of GO would adversely affect the crystal structure of UiO-66-NH_2_. When the GO content was 5%, UiO-66-NH_2_ was uniformly and regularly distributed on the GO surface, which facilitated the smooth passage of the complex components in cigarette smoke. The removal rate was 33.85% of UiO-66-NH_2_/GO 5%, which is 20.14% higher than that of UiO-66-NH_2_ and 25.54% higher than that of activated carbon. Overall, UiO-66-NH_2_/GO Y% had both the laminar structure of GO and the large specific surface area and π-π conjugation effect of UiO-66-NH_2_. The laminar structure of GO facilitated the passage of the complex components in cigarette smoke, and the large specific surface area and π-π conjugation effect facilitated the effective trapping of 1,3-butadiene in cigarette smoke.

In addition to 1,3-butadiene, the removal effectiveness for other components of the adsorbent materials was evaluated, such as some hazardous VOCs (benzene, toluene, isoprene) in cigarette smoke. The results (Table 5) showed that UiO-66 X% and UiO-66-NH_2_/GO Y% had different removal effects for the VOCs mentioned above. The removal effect of UiO-66-NH_2_/GO Y% was significantly higher than that of UiO-66 X%. Among those UiO-66-NH_2_/GO Y%, the UiO-66-NH_2_/GO 5% had the highest removal rates of 28%, 25%, and 23% for benzene, toluene, and isoprene, respectively, showing the important application of UiO-66-NH_2_/GO for the removal of hazardous components from cigarette smoke. The removal tendency for the VOCs mentioned above is basically consistent with that of 1,3-butadiene.

### 3.4. Adsorption of Pure 1,3-Butadiene

**Figure 7:**
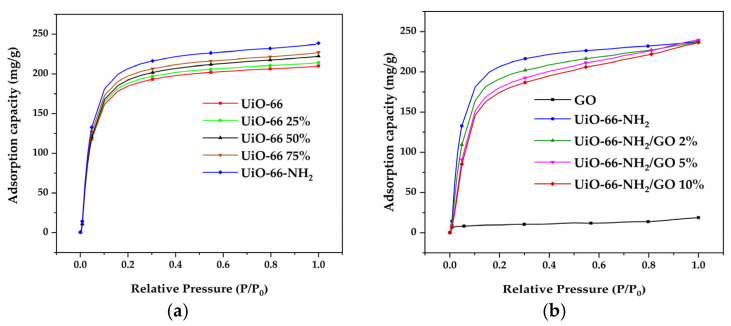
(**a**) Adsorption isotherm of UiO-66 X% on pure 1,3-butadiene. (**b**) Adsorption isotherm of UiO-66-NH*_2_*/GO Y% on pure 1,3-butadiene.

Figure 7 showed the adsorption isotherms of the adsorbent materials for pure 1,3-butadiene at 298 K, which presented the typical type I isotherm. The adsorption of 1,3-butadiene by the adsorbent materials significantly increased from relative pressure (P/P_0_) 0 to 0.2 and the adsorption slowly increased from relative pressure (P/P_0_) 0.2 to 1. Table 6 showed that the adsorption capacity of GO for 1,3-butadiene was only 18.79 mg/g, while the adsorption capacities of UiO-66 X% for 1,3-butadiene were as high as 210.01–238.68 mg/g and UiO-66-NH*_2_*/GO X% for 1,3-butadiene were as high as 236.46–239.54 mg/g. The adsorption capacities of the adsorbent materials for pure 1,3 -butadiene were way larger than that of 1,3-butadiene in cigarette smoke (1.28 mg/g). The first reason for this big difference was that the complex components of cigarette smoke had a negative influence on the adsorption of 1,3-butadiene. The second reason was the short interaction time (about 14 s) of adsorbent materials and cigarette smoke.

For UiO-66 X%, the saturated adsorption capacity of pure 1,3-butadiene gradually increased with the increase in NH_2_-BDC contents, which was consistent with the adsorption tendency of 1,3-butadiene in cigarette smoke. The saturated adsorption capacity of UiO-66-NH_2_/GO Y% on pure 1,3-butadiene was essentially consistent, which indicated that the introduction of GO did not affect the adsorption capacity of pure 1,3-butadiene. However, the introduction of GO significantly improved the ability to remove 1,3-butadiene from cigarette smoke, indicating that the main role of GO in cigarette smoke adsorption was to provide a laminar structure that facilitated the smooth passage of cigarette smoke.

## 4. Conclusions

A series of UiO-66 X% with different -NH_2_ contents was synthesized via the solvothermal method, and a series of UiO-66-NH_2_/GO Y% with different GO contents were prepared with controlling the ratio of UiO-66-NH_2_/GO. The introduction of -NH_2_ enhanced the interaction force between UiO-66 X% and 1,3-butadiene molecules and improved the removal of 1,3-butadiene from cigarette smoke, which implies the adsorption mechanism of UiO-66 X% on 1,3-butadiene including physical and chemical adsorption. The composites were obtained by introducing GO with a laminar structure, which facilitated the smooth passage of cigarette smoke components. Too little GO content in the composites resulted in UiO-66-NH_2_ densely accumulated on the surface of GO. Excessive GO contents in the composites resulted in UiO-66-NH_2_ being sparsely distributed on the surface of GO. When the GO content was 5%, UiO-66-NH_2_ was uniformly and orderly distributed on the GO surface and substantially improved the rate of 1,3-butadiene removal. In conclusion, the π-π conjugate action and the size constraint effect were the primary mechanisms for the adsorption of 1,3-butadiene molecules. The suitable interlayer channels facilitated the smooth passage of the complex components in cigarette smoke. The adsorption isotherms of pure 1,3-butadiene showed that the adsorption capacity of adsorbent materials for 1,3-butadiene was up to 210.01–239.54 mg/g, demonstrating that these adsorbent materials not only can be applied in reducing the release of 1,3-butadiene from mainstream cigarette smoke but also may have more potential applications in environmental protection.

## Figures and Tables

**Table 1:** Preparation parameters of materials.

Sample	ZrCl_4_ (g)	H_2_BDC (g)	NH_2_-BDC (g)	GO (g)	DMF (mL)
UiO-66	0.6991	0.4984	0	0	50
UiO-66 25%	0.6991	0.3738	0.1358	0	50
UiO-66 50%	0.6991	0.2492	0.2716	0	50
UiO-66 75%	0.6991	0.1246	0.4074	0	50
UiO-66-NH_2_	0.6991	0	0.5432	0	50
UiO-66-NH_2_/GO 2%	0.6991	0	0.5432	0.0140	50
UiO-66-NH_2_/GO 5%	0.6991	0	0.5432	0.0350	50
UiO-66-NH_2_/GO 10%	0.6991	0	0.5432	0.0699	50

**Table 2:** Structure parameters of UiO-66 X%.

Sample	BET (m^2^/g)	Pore Volume (cm^3^/g)	Pore Size (nm)
UiO-66	1135	0.43	0.81
UiO-66 25%	923	0.34	0.81
UiO-66 50%	847	0.32	0.81
UiO-66 75%	820	0.31	0.81
UiO-66-NH_2_	1088	0.40	0.81

**Table 3:** Structure parameters of UiO-66-NH_2_/GO Y%.

Sample	BET (m^2^/g)	Pore Volume (cm^3^/g)	Pore Size (nm)
GO	33	0.01	1.12
UiO-66-NH_2_	1088	0.40	0.81
UiO-66-NH_2_/GO 2%	928	0.35	0.83, 1.12
UiO-66-NH_2_/GO 5%	794	0.32	0.86, 1.06
UiO-66-NH_2_/GO 10%	766	0.29	0.91, 1.06

**Table 4:** 1,3-Butadiene removal efficiency in cigarette smoke.

Sample	1,3-Butadiene Yield (μg·cig^−1^)	1,3-Butadiene Removal Efficiency (%)
Blank control	38.21	0
Active carbon	34.95	8.31
UiO-66	33.87	11.15
UiO-66 25%	33.56	11.96
UiO-66 50%	33.40	12.38
UiO-66 75%	33.38	12.42
UiO-66-NH_2_	32.89	13.71
GO	35.42	7.06
UiO-66-NH_2_/GO 2%	27.93	26.73
UiO-66-NH_2_/GO 5%	25.21	33.85
UiO-66-NH_2_/GO 10%	27.39	28.14

**Table 5:** Other components removal efficiency in cigarette smoke.

Sample	Benzene (%)	Toluene (%)	Isoprene (%)
UiO-66	8.73	10.75	7.65
UiO-66 25%	9.29	10.06	7.94
UiO-66 50%	9.75	11.34	7.53
UiO-66 75%	9.62	11.27	7.89
UiO-66-NH_2_	10.72	11.65	8.35
GO	4.54	3.75	4.16
UiO-66-NH_2_/GO 2%	21.16	21.73	18.67
UiO-66-NH_2_/GO 5%	28.22	25.42	23.62
UiO-66-NH_2_/GO 10%	27.32	24.04	22.13

**Table 6:** Adsorption capacities of pure 1,3-butadiene on the adsorbent materials.

Sample	Adsorption Capacities of 1,3-Butadiene (mg/g)
UiO-66	210.01
UiO-66 25%	214.11
UiO-66 50%	222.46
UiO-66 75%	227.09
UiO-66-NH_2_	238.68
GO	18.79
UiO-66-NH_2_/GO 2%	238.01
UiO-66-NH_2_/GO 5%	239.54
UiO-66-NH_2_/GO 10%	236.46

## Data Availability

The data presented in this study are available on request from the corresponding author data.
